# Vas deferens to rete testis anastomosis for obstructive azoospermia

**DOI:** 10.1590/S1677-5538.IBJU.2024.0099

**Published:** 2024-04-07

**Authors:** Francesco C. Mesquita, Lucas R. Campos, Luis Felipe Savio, David Miller, Jason Codrington, Joshua Theodore White, David Velasquez, Akhil Muthigi, Ranjith Ramasamy

**Affiliations:** 1 University of Miami Desai Sethi Urology Institute Miami FL USA University of Miami, Desai Sethi Urology Institute, Miami, FL, USA; 2 Universidade Federal de Minas Gerais Departamento de Urologia Belo Horizonte MG Brasil Departamento de Urologia, Universidade Federal de Minas Gerais - UFMG, Belo Horizonte, MG, Brasil; 3 Hospital Beneficência Portuguesa de São Paulo Serviço de Urologia São Paulo SP Brasil Serviço de Urologia, Hospital Beneficência Portuguesa de São Paulo, São Paulo, SP, Brasil; 4 Houston Methodist Urology Associates Houston TX USA Houston Methodist Urology Associates, Houston, TX, USA

## Abstract

**Purpose:**

This video aims to present an in-depth, step-by-step tutorial on microsurgical reconstruction for obstructive azoospermia, featuring a distinctive case involving anastomosis from vas deferens to rete testis. The primary aim of this endeavor is to offer thorough and practical insights for healthcare professionals and researchers within the realm of reproductive medicine. The video endeavors to disseminate expertise, methodologies, and perspectives that can be advantageous to individuals grappling with obstructive azoospermia, providing a significant contribution to the progress of reproductive medicine and the augmentation of existing treatment alternatives.

**Materials and Methods:**

Surgical footage was recorded using the ORBEYE 4K 3D Orbital Camera System by Olympus America, with patient consent acquired for research purposes. Additionally, a retrospective examination of patient records was undertaken to compile relevant medical histories.

**Results:**

This video furnishes an exhaustive guide to microsurgical reconstruction for obstructive azoospermia, encompassing a distinctive instance of anastomosis from vas deferens to rete testis. State-of-the-art technology, such as the ORBEYE 4K 3D Orbital Camera, heightens procedural transparency, accentuating the significance of advanced instrumentation. The ethical underpinning is emphasized by obtaining patient consent for footage utilization, and a retrospective chart review augments the repository of valuable patient data. This comprehensive approach serves as an invaluable reservoir of knowledge for medical professionals and underscores excellence in clinical and ethical healthcare research.

**Conclusions:**

Anastomosis from vas deferens to rete testis emerges as a viable surgical reconstruction alternative for obstructive azoospermia, particularly when confronted with non-dilated tubules within the epididymis.

## INTRODUCTION

Azoospermia, characterized by the lack of sperm in the ejaculate (verified through the examination of two centrifuged semen samples), is observed in 15% of men facing infertility. It stems from pre-testicular, testicular, or post-testicular factors and can be categorized as obstructive azoospermia (OA) or nonobstructive azoospermia (NOA) (1). Out of all azoospermia cases, obstructive azoospermia (OA), attributed to an obstruction in the excurrent duct, constitutes 40% (2), which may manifest at various points along the male reproductive tract, encompassing the rete testis, efferent ducts, epididymis, vas deferens, and ejaculatory duct. The OA is identified with regular spermatogenesis, normal follicular stimulating hormone (FSH), and a normal testicular volume (15-25mL per testis) in a physical exam.

The etiological factors contributing to OA encompass various conditions such as ejaculatory duct obstruction, Wolffian duct abnormalities, Young's syndrome, infections, iatrogenic injuries, and elective sterilization (3). Currently, Percutaneous Epididymal Sperm Aspiration (PESA) and Micro Epididymal Sperm Aspiration (MESA) are the treatments indicated for obstructive azoospermia, where there is a blockage in the epididymis. They are recommended in cases of post-vasectomy recanalization failure, epididymal obstruction, congenital anomalies, or prior surgical interventions. Both aim to extract sperm for use in assisted reproductive techniques, such as in vitro fertilization (IVF). The choice between PESA and MESA depends on the patient's conditions and the physician's expertise (4).

Vasal obstruction is characterized by increased intraluminal pressure resulting in micro-rupture and blockage of the delicate epididymal tubules. Obstruction secondary to previous vasal obstruction may occur in up to 60% of men 15 years after vasectomy (5).

The most effective approach to address blockages in the vas deferens or epididymis involves microsurgical reconstruction, specifically opting for vasovasostomy (VV) or vasoepididymostomy (VE) as appropriate. The preferred technique in these cases is the multilayer microdot VV method (6), and for VE, the utilization of longitudinal intussusception vasoepididymostomy (LIVE) techniques (7) is recommended when feasible. In deemed unreconstructed cases, viable sperm can consistently be looked into for utilization in the context of IVF coupled with intracytoplasmic sperm injection (ICSI).

Silber's theory suggests that spermatozoa may not need to traverse the epididymis to attain the necessary maturity for successful fertilization of an oocyte (8).

The reconstruction of the vas deferens to the rete testis was undertaken based on the hypothesis that this procedure could facilitate natural conception in the patient.

## MATERIAL AND METHODS

The surgical proceedings were captured utilizing the ORBEYE 4K 3D Orbital Camera System provided by Olympus America, with explicit patient consent obtained for research objectives. Furthermore, a retrospective analysis of patient records was conducted to gather pertinent medical backgrounds.

## RESULTS

A 36-year-old male seeks an evaluation for potential male factor infertility in the context of a 1.5-year-long attempt to conceive with his 30-year-old female partner. The patient reports a prior semen analysis (SA) indicating azoospermia, although he does not have the results available.

He denies any history of testicular exposure to chemicals, radiation, or toxins. He also denies experiencing high fever, epididymitis, orchitis, prostatitis, sexually transmitted diseases, or testicular trauma, except for moderate urethral trauma. There is no reported history of varicocele, testicular torsion, cryptorchidism, post-pubertal mumps, or family infertility. Patient A has not undergone testosterone replacement therapy (TRT) and has no personal history of pelvic/abdominal surgeries.

His wife has irregular menstrual cycles and awaits evaluation by a reproductive endocrinologist. She has no significant medical history and is not currently on any medications.

The hormone levels, including LH (3.4 mIU/mL), P (7.7 ng/mL), T (386 ng/dL), and FSH (6.9 mIU/mL), fall within normal ranges. Physical examination indicates bilaterally descended testes with sizes of 14 cc (right, soft) and 20 cc (left, hard), and no palpable varicocele. Vas deferens and epididymis are palpable bilaterally. Genitalia examination reveals a normal phallus without lesions, and the meatus is in its typical anatomical position. These findings provide insights into his reproductive health, aiding in understanding potential factors influencing the couple's difficulty in achieving pregnancy.

His assessment confirms azoospermia. Genetic testing has returned normal results. A follow-up is scheduled post-ultrasound, with a contingency plan for Testicular Sperm Aspiration (TESA) if the ultrasound shows no abnormalities. This streamlined approach aims to efficiently address the identified issues in male fertility and inform targeted interventions.

TESA indicated obstructive azoospermia. During discussions about treatment options, the costs associated with primary VE versus sperm retrieval for IVF were explored. Considering the financial considerations, the patient has expressed a preference for proceeding with primary VE. The plan included freezing sperm at the time of the procedure for potential future use.

The procedure used the Orbeye 4k 3D. Note that the vasal fluid appeared clear and no sperm were identified. A 24-gauge Angiocath was placed into the vasal lumen and normal saline was injected to allow examine patency of the abdominal vas. We decided to proceed with VE. The vas deferens were dissected out. A 5-0 chromic suture was placed in the perivasal tissue as a holding stitch on the testicular site. The vas was then transected using Dennis Blade. Hemostasis was achieved using bipolar electrocautery. The vasal segment was tied off with the 5-0 chromic suture and bovie electrocauterized. Attention was then turned to the abdominal vas. The abdominal vas was blunted dissected to external ring and mobilized in order to achieve adequate length and reapproximate the vas to the epididymis. A 5-0 chromic perivasal holding suture was placed. Good hemostasis was achieved. The tunica vaginalis was incised and opened with dissection and care was taken to protect the epididymis and vasal structure. The epididymis was carefully inspected and examined; however no dilated tubules were found in both sides. The level of obstruction was not readily apparent, then a 10-0 needle was used to puncture an epididymal tubule initiating the process distally and advancing, toward the proximal end (up to the caput), checking for sperm presence in the fluid under the microscope after each puncture. Three attempts were made on each side, but no sperm was found. As a last attempt, and already described in the literature (9), a new dissection was carried out, this time in the rete testis, and dilated tubules were visualized. In preparation for the double arm intussusception technique, two 10-0 nylon double arm sutures were placed in parallel along the sides of the rete testis tubules. Note, these sutures were left in place, not pulled through. The tubule was incised, and the fluid appeared cleary. Microscopic exam revealed many motile sperms bilaterally. Several 9-0 nylon sutures were placed through the outer layer of the vas to the epididymal tunic to further reapproximate the anastomotic edges. After that, the pre-placed 10-0 sutures in the rete testis were placed at the mucosa of the inner layer of the vas, and a near-near far-far technique to intussuscept the vas into the rete testis. An excellent approximation of the inner and no liquid identified. After that, the outer muscular layer of the anastomosis was approximated to the remaining tunica, using interrupted 9-0 nylon sutures, completing the anastomosis.

The informed consent document was administered to the patient in accordance with scientific protocols.

## DISCUSSION

We are dealing with a case of obstructive azoospermia, where initially we considered reconstruction through VE. However, we encountered the absence of dilation in the tubules of the epididymis, rendering this approach unfeasible. Faced with this situation, we opted to perform an anastomosis between the vas deferens and the rete testis.

This alternative, supported by the theory that spermatozoa may attain the necessary maturity for successful fertilization of an oocyte without traversing the epididymis, provides a viable chance of achieving a natural pregnancy for the patient (8). In typical cases of obstructive azoospermia where VV or VE is not possible, the usual recourse is to turn to IVF or ICSI. However, the choice of anastomosis in the rete testis was made due to the potential success of the procedure and the consideration of costs associated with more complex treatments, such as IVF or ICSI.

It is important to highlight that, even adopting this approach, we collected sperm for cryopreservation. This measure aims to ensure the availability of sperm for potential assisted reproductive procedures in the future. We aim to provide the patient not only with an immediate solution but also the security of future options.

We believe that this specific approach was carefully weighed, taking into account the patient's best interests, the risks involved, and the pursuit of an effective and sustainable solution for their unique situation.

## CONCLUSION

The consideration of vas-to-rete-testis reconstruction becomes particularly pertinent in cases where VE is indicated, and there is an absence of tubular dilation in the epididymis. The absence of such dilation underscores the relevance of this reconstructive procedure, emphasizing its suitability in specific contexts of male infertility. The positive outcomes associated with this approach, coupled with its financial advantage over more complex alternatives like IVF and ICSI, further support its consideration as a valuable option for patients facing these specific reproductive challenges.

**Available at:** http://www.intbrazjurol.com.br/video-section/20240099_Ramasamy_et_al

Int Braz J Urol. 2024; 50 (Video #4): 368-72

## References

[1] Esteves SC, Miyaoka R, Agarwal A (2011). An update on the clinical assessment of the infertile male. [corrected]. Clin Sao Paulo Braz.

[2] Practice Committee of American Society for Reproductive Medicine in collaboration with Society for Male Reproduction and Urology (2008). The management of infertility due to obstructive azoospermia. Fertil Steril.

[3] Goldstein M (1985). Microspike approximator for vasovasostomy. J Urol.

[4] Kumar R, Mukherjee S, Gupta NP (2010). Intussusception Vasoepididymostomy With Longitudinal Suture Placement for Idiopathic Obstructive Azoospermia. J Urol.

[5] Wosnitzer MS, Goldstein M (2014). Obstructive Azoospermia. Urol Clin North Am.

[6] Shah R (2011). Surgical sperm retrieval: Techniques and their indications. Indian J Urol IJU J Urol Soc India.

[7] Baker K, Sabanegh E (2013). Obstructive azoospermia: reconstructive techniques and results. Clin Sao Paulo Braz.

[8] Silber SJ, Balmaceda J, Borrero C, Ord T, Asch R (1988). Pregnancy with sperm aspiration from the proximal head of the epididymis: A new treatment for congenital absence of the vas deferens. Fertil Steril.

[9] Weiske WH (1994). Pregnancy caused by sperm from vasa efferentia. Fertil Steril.

